# Raw diffraction data and reproducibility

**DOI:** 10.1063/4.0000232

**Published:** 2024-02-14

**Authors:** Loes M. J. Kroon-Batenburg, Matthew P. Lightfoot, Natalie T. Johnson, John R. Helliwell

**Affiliations:** 1 Crystal and Structural Chemistry, Bijvoet Center for Biomolecular Research, Utrecht University, Universiteitsweg 99, 3584 CG Utrecht, The Netherlands; 2 Cambridge Crystallographic Data Centre, 12 Union Road, Cambridge CB2 1EZ, United Kingdom; 3 Department of Chemistry, University of Manchester, Manchester M13 9PL, United Kingdom

## Abstract

In recent years, there has been a major expansion in digital storage capability for hosting raw diffraction datasets. Naturally, the question has now arisen as to the benefits and costs for the preservation of such raw, i.e., experimental diffraction datasets. We describe the consultations made of the global structural chemistry, i.e., chemical crystallography community from the points of view of the International Union of Crystallography (IUCr) Committee on Data, of which JRH was the Chair until very recently, and the IUCrData Raw Data Letters initiative, for which LKB is the Main Editor. The monitoring by the CCDC of CSD depositions which cite the digital object identifiers of raw diffraction datasets provides interesting statistics by probe (x-ray, neutron, or electron) and by home lab vs central facility. Clearly, a better understanding of the reproducibility of current analysis procedures is at hand. Policies for publication requiring raw data have been updated in IUCr Journals for macromolecular crystallography, namely, that raw data should be made available for a new crystal structure or a new method as well as the wwPDB deposition. For chemical crystallography, such a step requiring raw data archiving has not yet been recommended by the IUCr Commission on Structural Chemistry.

## I. INTRODUCTION

Olga Kennard founded the Cambridge Crystallographic Data Center (CCDC) at the University Chemical Laboratory in Cambridge in 1965. She started compiling crystal structures in the “Molecular Structures and Dimensions” series that were published in the 1970s and 1980s and were filling many bookshelves in crystallography labs all over the world. This classified bibliography of organic and organometallic crystal structures covered the period 1935–1983. Our knowledge of bond distances (later used in refinement as additional observations) is largely a result of her efforts. She had the vision that compiling results from different, individual experiments in one place would open doors to new insights and knowledge (https://www.ccdc.cam.ac.uk/discover/blog/celebrating-ccdc-founder-and-crystallographer-dr-olga-kennards-iucr-ewald-prize/). The Cambridge Structural Database (CSD) now covers all these data and has continued building up the collection with now over 1.2 million precise 3D structures with data from x-ray, neutron, and electron diffraction, and in addition, the CCDC provides many analysis tools. Since 2011, the CCDC began accepting experimental structure factor data alongside crystallographic information files (CIFs) for deposition of structures to the CSD, following the IUCr Journals policy to require structure factor data for published crystal structures as part of the checkCIF procedure (Spek, 2003).

In data archiving, data reuse is an important measure of utility. There are various aspects to this for the CCDC. For the past 3 years, over 90% of structures submitted to CCDC have either accompanying or embedded hkl/fcf information available, and it is the default setting for users to download these data from the CCDC's online Access Structures/Web CSD Service (Thomas *et al.*, 2010). While it is not possible to track how frequently structure factors are downloaded and reused, there are several examples of data re-use in the CSD, such as where existing structures have been re-refined using techniques beyond the independent atom model (Woińska *et al.*, 2023; Sanjuan-Szklarz *et al.*, 2020; Jha *et al.*, 2020; and Woińska *et al.*, 2016). The CCDC also re-uses structure factor information internally in their data integrity checks. To ensure acknowledgment of the original data, the CCDC applies cross-references (visible in the Desktop CSD software) to structures, which use the same experimental reflection data, when aware of it. The CCDC also recommend that authors of any paper using existing data cite the original paper and the CCDC data in their articles. There are currently around 1000 re-refinements in the CSD; however, some of these are due to different disorder models of the same data rather than separate uses of existing data.

In response to a call from the crystallographic community for preserving and sharing raw diffraction data (Kroon-Batenburg *et al.*, 2017; Helliwell *et al.*, 2017) and following the FAIR data principles (Wilkinson *et al.*, 2016), the CCDC allows depositors to provide a persistent link (DOI) to where their raw data can be found. Thus, data entries in the CSD can now cover the complete range of data (coordinates/processed data/raw data) resulting from a single crystal structure determination. In this paper, we discuss the need for reproducibility in science, and the role that raw data archiving and the FAIR data principles can play in this. These opportunities for raw diffraction data archiving raise further questions about their reuse utility such as: How much raw data are likely to be reused (and, thus, how does deposition of raw datasets figure in a cost/benefit analysis)? These are difficult to answer at this early stage. However, Helliwell *et al.* (2017) explored this aspect via case studies in a range of crystallographic areas, including chemical crystallography. Since then, artificial intelligence and machine learning (AI and ML) has grown considerably in various ways. We expect then that machine learning techniques will find a big role with many raw datasets becoming archived and thereby available. We expect ML to be applied to various aspects of the raw diffraction data processing steps: like reflection indexing, identification of multiple lattices, mosaicity estimation, the presence of satellite reflections, or diffuse scattering. In the paper of Helliwell *et al.* (2017), examples are also given of cases where availability of raw diffraction data would have helped in correcting errors in published structures. An additional example in chemical crystallography is recently published by Lutz (2023).

## II. METADATA ARE A CRITICAL ASPECT OF REPRODUCIBILITY

Kennard (1967) set out the critical metadata for communicating crystal structures. As their brief abstract describes, they provided:

“A report containing a list of recommendations on the presentation of crystallographic data in primary publications relating particularly to single-crystal work. The more important items of information are discussed in detail with examples. Numerical values of certain constants in common use are recorded.”

In this article, instead of reproducibility, we find the word “validity” of a crystal structure. In particular, it is stated that:

“It is also customary to give a table of observed amplitudes |Fo| and calculated structure factors |Fc|. There are strong arguments in favour of this procedure: (a) The table is the ultimate evidence for the validity of the analysis.”

Olga Kennard was the first of the IUCr's Representatives to CODATA. In her first report to the IUCr Executive Committee (1968), she wrote:-

“Committee on Data for Science and Technology (CODATA) of the International Council of Scientific Unions

The Representative of the Union, Mrs. O. Kennard, attended the third meeting of CODATA, held in Frankfurt/Main, B.R.D., in June 1968, and the subsequent First International CODATA Conference at Arnoldshain. She gave a report on the activities of the Union and contributed to the discussion on the role of Scientific Unions in the field of data and documentation. At both meetings, the future of CODATA and its relation to a world-wide information network were discussed.”

**FIG. 1.**
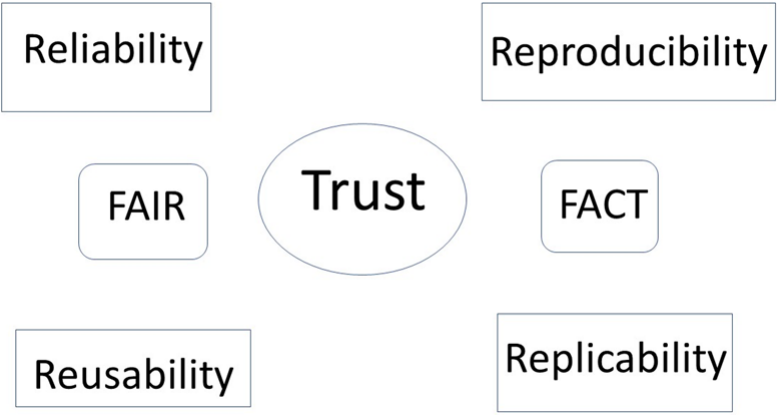
Trust in science is built up from different facets. Reproduced with permission from Helliwell and Massera, J. Appl. Cryst. **55**, 1351–1358 (2022). Copyright 2022 IUCr Journals.

The major role of CODATA in global science has continued; JRH was the IUCr's Representative from 2012 to 2023. In that role, we note that various aspects of “reproducibility” of any study, across all the sciences, was and is seen as vital for ensuring the trust in science. Helliwell and Massera (2022) described these across the scientific fields’ terminologies, from which Fig. 1 is shown again here.

## III. CONSULTATION OF THE STRUCTURAL CHEMISTRY GLOBAL COMMUNITY OF THE POTENTIAL OF RAW DIFFRACTION DATA ARCHIVING

In 2018, Simon Coles and Amy Sarjeant started a consultation of the global structural chemistry community principally via the announcement of a questionnaire in the IUCr Newsletter (Coles and Sarjeant, 2018) of the potential of raw diffraction data archiving in chemical crystallography. One of us (JRH) wrote a Foreword to their article, stating that crystallographers have a strong tradition of linking data to publications, with chemical crystallography using technologies like crystallographic information files (CIF) and checkCIF procedures. Also, that *Acta Crystallogr., Sect. C* exemplifies this with its submission process, and that the need for preserving raw diffraction datasets, recommended by the IUCr's Diffraction Data Deposition Working Group, aligned with the FAIR principles (Findable, Accessible, Interoperable, Reusable) for scientific data. Coles and Sarjeant (2020) observed that case studies across biological and chemical crystallography and powder diffraction were published to demonstrate the value of preserving raw data (Helliwell *et al.*, 2017).

From the Questionnaire returns, in terms of need, Coles and Sarjeant (2020) summarized that it was in the domain of the advanced techniques that the strongest need was perceived to exist for robust raw data management, validation and sharing—although there would, of course, be commonalities with some of the tougher service crystallography examples such as disordered and incommensurate structures. In all of these cases, they observed that the drivers for sharing raw diffraction data in chemical crystallography would be to generate further insight—or that future methods might be able to extract more information without the need to repeat the experiment.

**FIG. 2.**
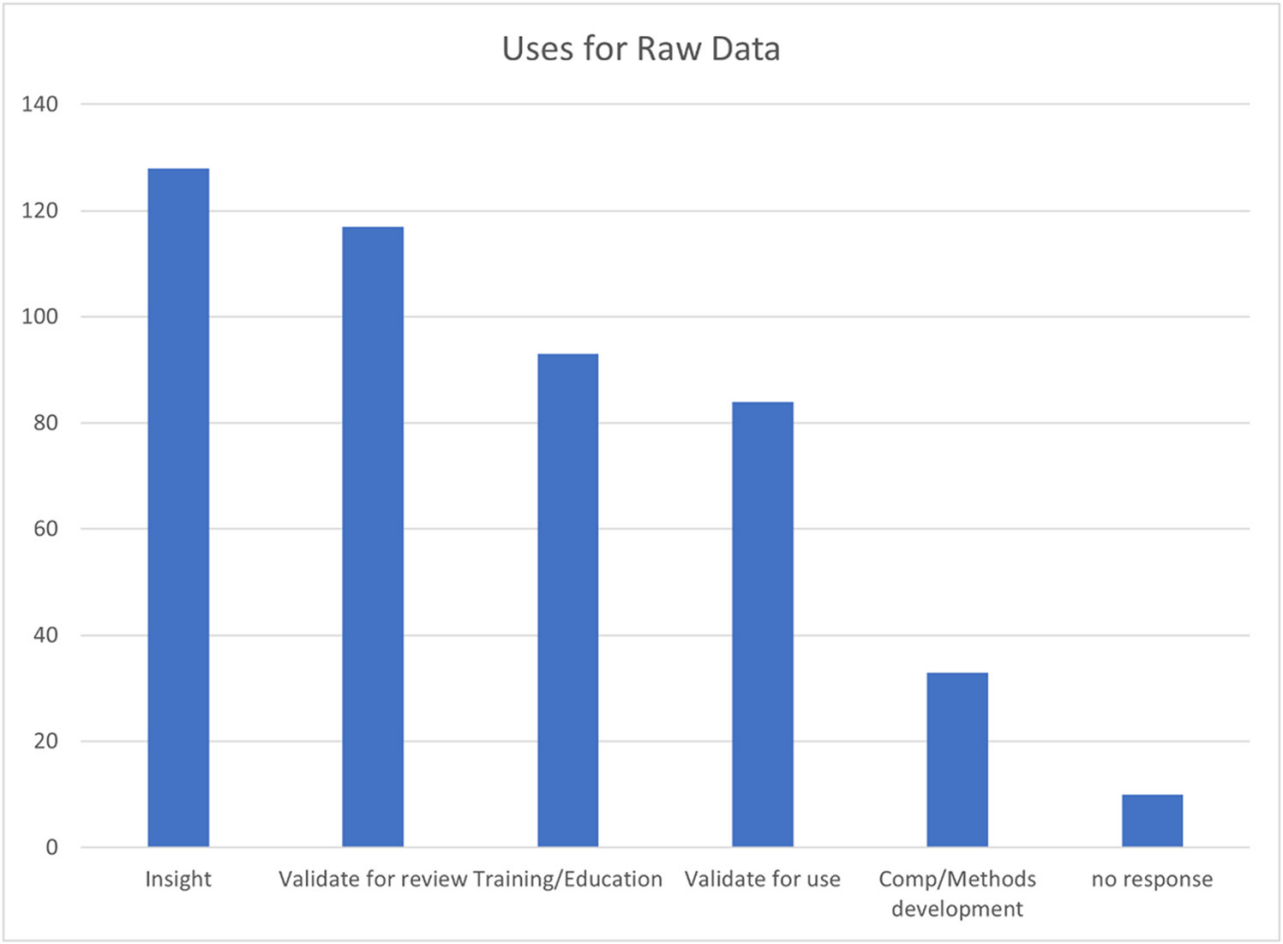
The benefits of having access to raw data, as identified by the survey respondents. Reproduced with permission from Coles and Sarjeant, see https://www.iucr.org/news/newsletter/volume-28/number-1/raw-data-availability-the-small-molecule-crystallography-perspective for “IUCr Newsletter (2020).” Copyright 2020 IUCr Journals.

Also, they highlighted via a histogram the insights offered by the questionnaire responders (Fig. 2).

This was followed up by holding a Workshop by Zoom under the auspices of the IUCr Congress in Prague (Coles and Sarjeant, 2021). The talks at the Workshop spanned chemical crystallography structure determination facility providers, home lab, and central facility as well as special categories such as modulated/incommensurate structures, high pressure chemical crystallography, difficult data cases, and quantum crystallography studies. These special cases challenged the crystallographer either due to relatively low diffraction data completeness (high pressure) or different raw diffraction data software not having been tested with a particular difficult case (e.g., smeared diffraction spots and poor diffraction resolution).

After this consultation of the global chemical crystallography community, via questionnaire and workshop, and at the encouragement of the IUCr Commission on Structural Chemistry, the raw data archiving needs were further presented at the IUCr 2023 Congress Committee on Data Workshop (Kroon-Batenburg *et al.*, 2023; Britten, 2024; Coles, 2024).

Coles (2024), in particular, observed that exciting advances (such as electron crystallography or dynamic crystallography) must be set against the backdrop of traditional x-ray crystal structure analysis, with its >100 years of enhancing instrumentation, >50 years of collecting results into databases, 40+ years of trusted common refinement processes, 30 years of standards, and 20+ years of validation tools. Therefore, the small-molecule crystallography community now caters very well with its processed diffraction data and molecular atomic models for the validation and quality control of relatively routine structures as part of the checking and publication process. New procedures will likely be needed for the new methods and the very challenging chemical crystal structures, and, therefore, that the raw diffraction data should in those special cases also be preserved. Related to this, at the same Workshop, Britten (2024) noted that diffraction space is more complicated than we often imagine and contains unmined information about our samples. Scattering from aperiodic crystals is an obvious example. The value of preserving raw 3D diffraction data cannot be underestimated. Resonating with this is that some opinions expressed at the IUCr Workshop, in Prague and in Melbourne, were that quantum crystallography should be more widely implemented, and raw diffraction data preservation could expedite that. Also, Henn (2019) has advocated adding new metrics to chemical crystal structure validation, which might be usefully expanded to reprocessing preserved raw diffraction data. This connects with the long-standing issue of “fitness for purpose of a given crystal structure determination” whereby a molecular structure may be determined solely for the purpose of chemical characterization and thereby to a lower degree of precision than the crystal sample itself can offer. Since an archive of crystal samples is not really practical, the preservation of raw diffraction data in any given study is the next best option and would allow those data to be re-processed. Indeed, some crucial information may be lost in reducing the raw data to the processed structures factors, e.g., relating to symmetry, multiple lattices (neglected overlap of Bragg reflections), and additional features such as diffuse scattering. In addition to a high-quality crystal case, a low-quality crystal is often apparent from the raw diffraction images, and it may need the skills of a crystallographic expert to extract all information.

So, with this strong chemical crystallography community interest in raw diffraction data archiving in the specific categories listed above, what developments are evident? The CCDC has been monitoring this, and the details are described in Sec. IV.

## IV. MONITORING OF RAW DIFFRACTION DATA DEPOSITIONS AT THE CCDC

**FIG. 3.**
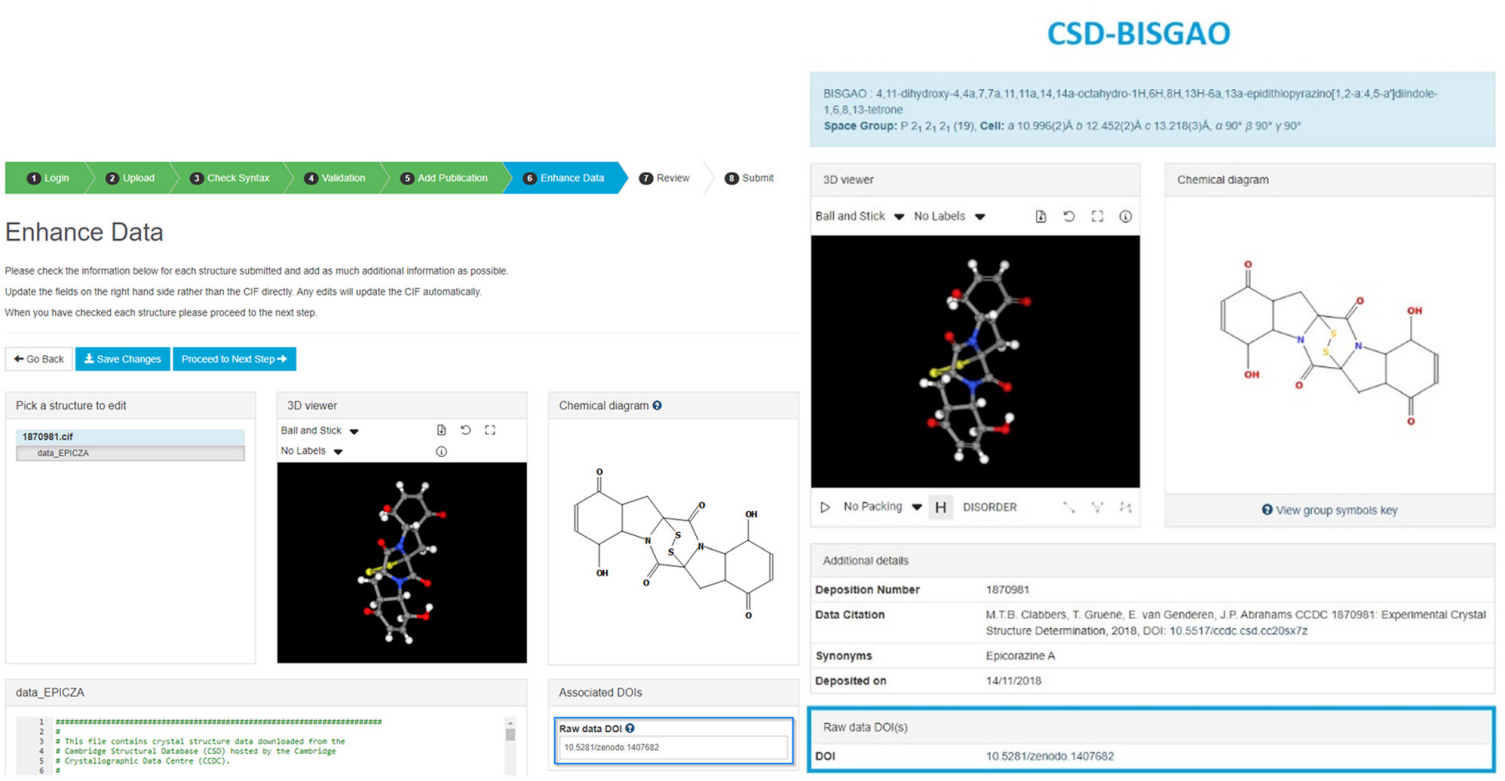
The procedure used by the CCDC (left) and an example of raw diffraction data archiving (right) of which data can be found at https://dx.doi.org/10.5517/ccdc.csd.cc20sx7z.

**FIG. 4.**
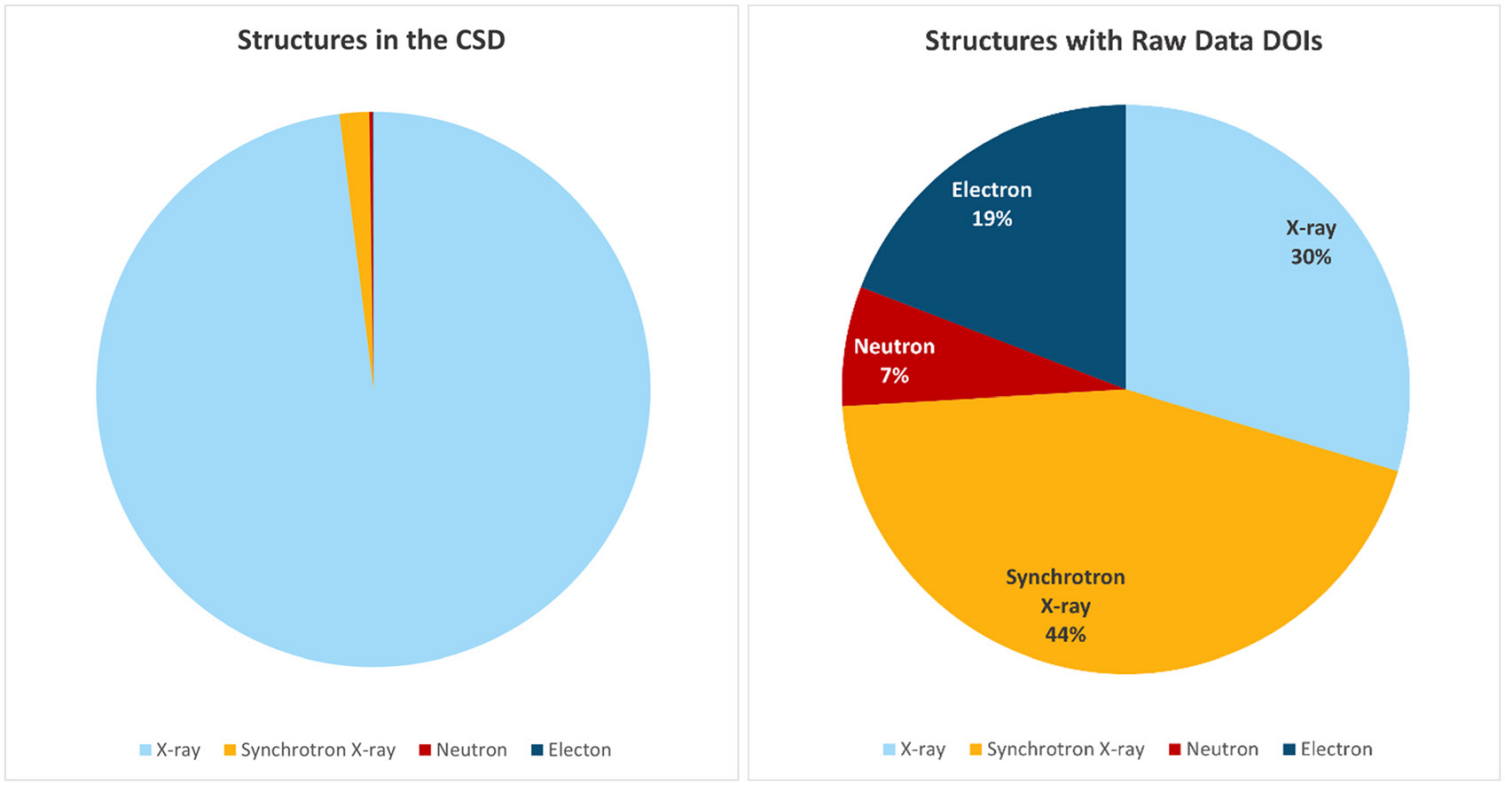
The portion of structures with raw data DOIs and diffraction probes used.

**FIG. 5.**
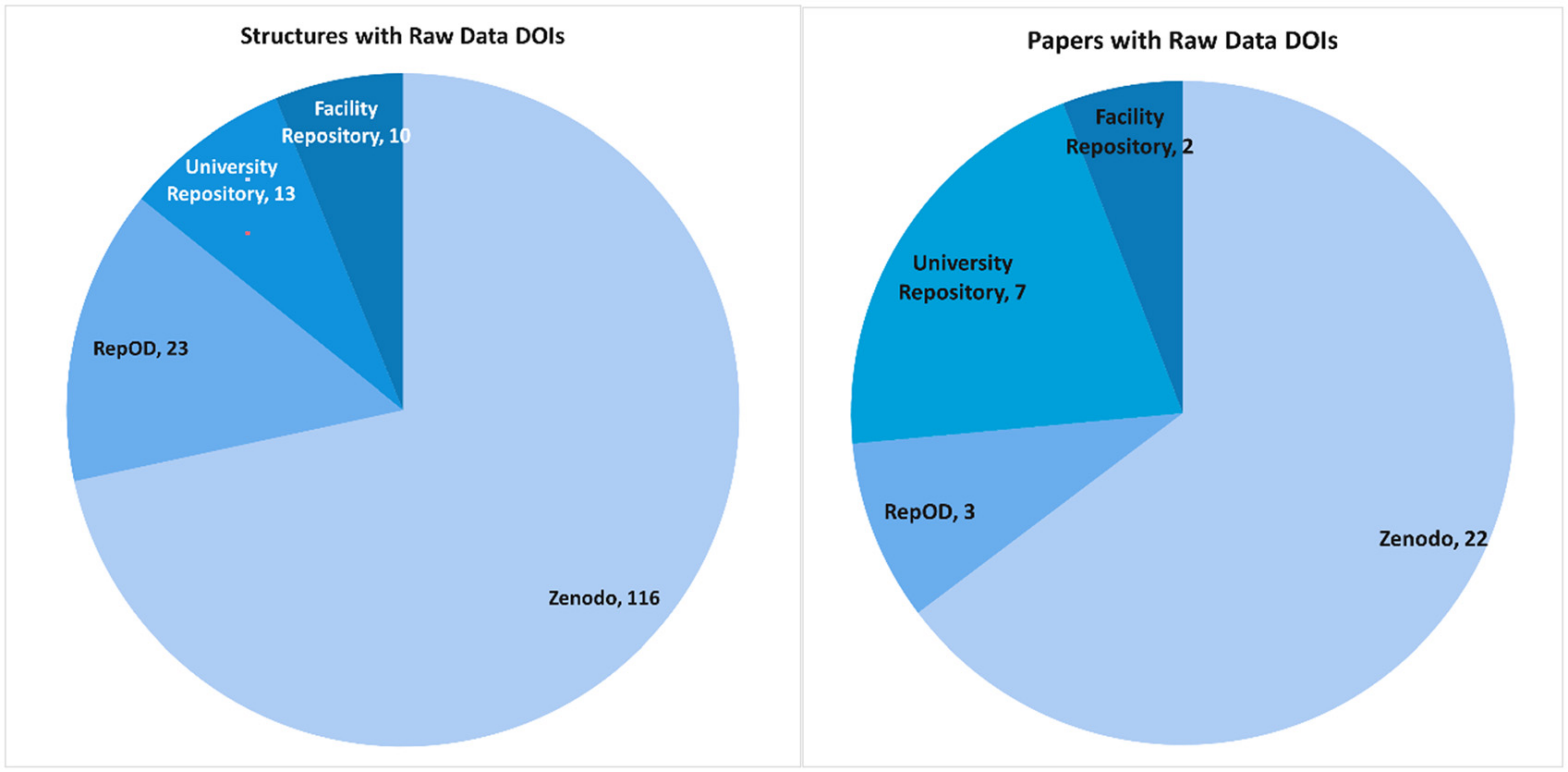
In total, 162 structures have raw data, distributed over the various data repositories as shown on the left. The number of papers having raw data DOIs is shown on the right.

The CCDC introduced the possibility for a depositor into the CSD to log a digital object identifier (DOI) to an archived raw diffraction dataset if they wished. Below, we present details of the instances so far of raw diffraction data DOIs (Figs. 3–5).

The CSD currently has >1.2 million entries. So, the 162 structures from 34 different papers are a small percentage (0.01%). However, impressive to us as authors of this article is that there are a wide range of repositories available now (Fig. 5). Considering the various consultations made by Coles and Sarjeant (2018; 2020; 2021), the statistics made available by the CCDC (Figs. 3–5) are a good match so far. A FAQ on raw data DOIs is available at the CDCC website (https://www.ccdc.cam.ac.uk/support-and-resources/support/case/?caseid=e1fc6d58-e3b7-e711-b787-005056977c87).

## V. RAW DATA LETTERS INITIATIVE

Chemical crystallographers are used to depositing coordinates and structure factors with their publications and to the CSD but may still be uncomfortable with making their raw diffraction data available. A recent paper by Kroon-Batenburg (2023) gives guidelines and instructions of how to publish raw data to Zenodo for Macromolecular crystallography. The guidelines are also applicable to chemical crystallography, but a few words have to be said about data formats and detector calibrations. The data are often collected at a home diffractometer, which have manufacturer-developed detectors and sometimes proprietary undisclosed binary image formats. However, these issues do not need to hamper raw data publication if we follow the guidelines given below.

IUCrData Journal has launched a new section, Raw Data Letters (Kroon-Batenburg *et al.*, 2022), and is a collaborative innovation of IUCr Journals with the IUCr Committee on Data (CommDat). The aim is to publish short descriptions of crystallographic raw datasets from x-ray, neutron, or electron diffraction experiments, in the biological, chemical, materials science, or physics fields, and provide a persistent link, preferably a DOI, to the location of the raw data. The letters will describe interesting features in raw datasets, allowing researchers to attract attention to particular aspects of the data. This allows methods and software developers to devise improved methods for (re)analysis of the data or experts to extract more structural details from the data. Raw Data Letters follow the FAIR data guidelines. A big concern in publishing raw data is the re-usability, which requires that the data come with accurate and complete metadata and well-described image data formats. A project team supporting Raw Data Letters first established a core metadata list, then developed tools to extract metadata from image headers, and write these to an imgCIF file. These will be checked with a CheckCIF tool, which runs on the IUCr webserver, to verify the consistency and correctness of the metadata data (https://iucrdata.iucr.org/x/services/rawdataletters.html). The accessibility of all repositories referenced by archived imgCIF files held by the journal will be regularly checked to avoid problems with no longer existing landing pages. The project team is working with the diffraction equipment vendors to solve (meta)data formats to facilitate exporting raw data and removing barriers to raw data publication.

Two recent Raw Data Letters in Chemical crystallography may serve as a guideline of how to write such papers. The first paper (Lutz and Kroon-Batenburg, 2022) describes the twinned γ-form of o-nitroaniline. The twinning results in stacking faults in hydrogen bonded layers and streaked diffuse scattering. The second paper (Bernès and Camargo, 2023) describes a new structural model for the disorder in the cyclohexane plastic phase I and associated diffuse scattering.

## VI. THE FUTURE

Overall, the likely future for raw diffraction data in chemical crystallography has been carefully researched by Coles and Sarjeant (2018; 2020; 2021).

These authors have also kept a scrutiny of the equivalent practice in macromolecular crystallography. The IUCr Commission on Biological Macromolecules, led by Dr. Wladek Minor, has engaged firmly with the IUCr Journals, supported by the IUCr Committee on Data, to effect a formal inclusion of the need for future articles in biological crystallography in IUCr Journals to “require raw diffraction data only where a new structure was reported or a diffraction data processing software was reported” (Helliwell *et al.*, 2019). The IUCr Commission on Structural Chemistry will be consulted again when the outcomes of the IUCr 2023 Workshop are published (Kroon-Batenburg *et al.*, 2023).

Clearly, raw diffraction data as part of the formal scientific version of record of a given study have firmly entered the arena of structural science. There are two aspects that will open up new opportunities. First, the tracking of different workflows arising from a raw diffraction dataset as the “ground truth,” i.e., to borrow a term from machine learning procedures, is largely unexplored or at least unpublished, and what is their impact on atomic coordinate errors and atomic displacement parameters (ADP) (Helliwell, 2023)? There are likely to be situations for real system materials with challenging diffraction patterns. These are often powder diffraction cases. The issues of raw powder diffraction data are relatively unexplored but again offer new opportunities. A case study from the ICDD (Reid *et al.*, 2016) is highlighted in Helliwell *et al.* (2017). Also, an analysis of powder diffraction raw data archiving and reuse is provided by Aranda (2018). Second, machine learning techniques have great potential to explore the future archives of both published and unpublished raw datasets. These techniques will require enabling machine readability of raw diffraction data. An excellent start to such machine-based approaches has been made by IUCrData Raw Data Letters' project team with its checkcif for raw data. This ensures the readability/reuse of a raw diffraction dataset by evaluating key aspects such as whether the beam center of the diffraction images is accurately known.

Finally, we again quote Olga Kennard: “I think that the great ocean of truth is still in front of us and that we will continue to discover new aspects of this truth” [quoted by Dr. Suzanna Ward in her lecture at IUCr 2023 Congress Melbourne (Ward, 2023)]. Raw diffraction data archiving is in its infancy, rather than maturity, and its benefits for deeper truths in future in its re-processing will have to unfold. We imagine that Olga Kennard would study these matters closely.

## Data Availability

The data that support the findings of this study are available within the article.
